# Perceived climate change impacts on food security in coastal communities of Puerto Princesa City, Philippines

**DOI:** 10.14324/111.444/ucloe.3512

**Published:** 2026-05-06

**Authors:** Karen G. Madarcos, Lutgardo B. Alcantara, Lota A. Creencia

**Affiliations:** 1 College of Public Administration and Management, Western Philippines University, Aborlan, Palawan, Philippines; 2 College of Fisheries and Natural Sciences, Western Philippines University, Sta. Monica, Puerto Princesa City, Palawan, Philippines

**Keywords:** climate change, food availability, vulnerability, food security, coastal communities, Palawan

## Abstract

The Philippines is one of the most vulnerable archipelagic low–middle-income countries in regard to the impacts of climate change. Consequently, Puerto Princesa City, a coastal city in central Palawan, Philippines, faces the challenges of these impacts, which affect the food security of its coastal communities. This research presents an assessment of perceived climate changes and investigates their impact on food security. The research employed descriptive analysis to assess the perception of the residents towards climate change and its impacts, and multiple linear regression to examine the connections between climate change indicators and the fundamental components of food security within coastal communities. The results revealed that most participants (94%) believe climate change is happening, and many (71%) acknowledged this as anthropogenic. There are observations of sea level rise (76%), wave intensity (69%), warmer sea surface temperature (73%), and more frequent and stronger rainfall (72%) in comparison to 10 years ago. Coastal communities have become less food secure. Sea level rise was significantly associated with decreased food availability, access and stability (*p* < 0.05). Participants’ perception of extreme rainfall events and increased sea surface temperatures were associated with reduced food utilisation, leading to increased exposure to infectious diseases, pollution along the shores, and decreased fish growth and stock in the usual fishing spots (*p* < 0.05). This study provides valuable insights into the perceptions of climate change and its impacts on food security in coastal communities and highlights the necessity to understand food security in the Philippines and other low–middle-income countries vis-à-vis climate change and integrate holistic measures into the local and national agenda to mitigate the associated risks.

## Introduction

The United Nations defined climate change as ‘long-term shifts in temperatures and weather patterns’. This change is caused by natural and anthropogenic phenomena [1] and could be more profound in poor and developing, low-lying and hot countries [2] compared to richer ones. The Philippines, classified by the World Bank as a low–middle income country (LMIC), with a coastline spanning up to 36,289 km, located just above the equator, is strongly influenced by these three factors, making it one of the most vulnerable countries to the impact of a changing climate. The country ranked third most at risk of climate change and has the highest sensitivity to extreme events across 67 countries globally [3]. It is exposed to climate-related hazards such as increased frequency of natural disasters, extreme rainfall, increased sea surface temperatures (SST) and sea level rise (SLR). The combined effects of these hazards pose a serious threat to the food and nutrition security of the poor and vulnerable coastal communities [4].

Nations across the world aim to increase the quality of life by increasing food security. However, climate change slows collective progress towards this goal [5], more so in countries more vulnerable to malnutrition and food insecurity [6]. Food security is ‘when all people, at all times, have physical and economic access to sufficient safe and nutritious food that meets their dietary needs and food preferences for an active and healthy life’. It has four interrelated key dimensions: availability, access, utilisation and stability. Availability addresses food supply, including domestic production and imports. Access is determined by both physical and economic means to acquire the food supply. Utilisation pertains to the way food is consumed, while stability refers to the consistency and reliability of the three other pillars.

Based on the Food and Agriculture Organisation data, the IBON Foundation [7] reported that the Philippines had the worst incidence of severe and moderate food insecurity in Southeast Asia in 2024 translating to almost 51 million severely and moderately food insecure people. This food insecurity is propelled by poor amount and quality of food and is compounding severe malnutrition [8]. Only 45% of the population can afford food adequate for the energy and nutrient requirements of a usual household [9]. The rest has comparatively less fruit, milk, other dietary products, and fish and meat on the table, resulting in less energy and nutrient intake. Farmers and fishers, and those who live in rural areas were among the least food secure in the country [9]. Coastal communities are also among the poorest of the poor [10]. For fishers living on the coasts, the ocean is the primary source of fishery-related livelihood and food security. However, the changing climate will alter these coastal areas and their related services [11].

Coastal communities around the world are facing unprecedented challenges from climate change, including rising SST, SLR, shifts in precipitation patterns and intensity, and changing wave strengths. These impacts pose a significant risk to food security, water resources, human health, fisheries and human settlements. Furthermore, those who reside in these poor coastal communities will carry the weight of the additional burden that climate change is expected to bring, paralysing their ability to strive for a better quality of life.

Studies on climate change that have affected food security have been conducted by government agencies and academes in various parts of the Philippines. However, there is little known study on the changing climate felt near the coast and how these changes affect coastal communities’ perceived food security. Studying coastal communities should be at the forefront of climate change research because their proximity to the ocean enables them to have a stronger belief in climate change [12].

To better understand the impacts of climate change on the food security of coastal communities, our study takes a community-based perception approach. By evaluating climate hazard impacts from the perspective of those directly affected, we hope to gain a more nuanced understanding of the challenges faced by these communities. Therefore, this study investigates the coastal communities’ (1) awareness of climate change and its risks; (2) their perception of the level of their food security; (3) characteristics that influence their awareness and perception of climate change and food security; and (4) how their climate change awareness impacts their perceived level of food security.

## Materials and methods

### Study area and sample

Our study focuses on Puerto Princesa City, the second largest city in the Philippines in terms of land area, located 306 nautical miles southwest of Manila, the country’s capital. There are 18 declared fish/marine sanctuaries in the city, and a significant portion of households are engaged in fishing. With a coastline that extends up to 416 km and municipal waters covering an area of 27,586 ha, 74% of its barangays (villages) are in the coastal areas [13]. Eleven of the declared fish/marine sanctuaries are located on the east coast, particularly Honda Bay, while the remaining seven are in the west and northwest, covering 7.65% of the city’s declared municipal waters [14].

**Figure 1:**
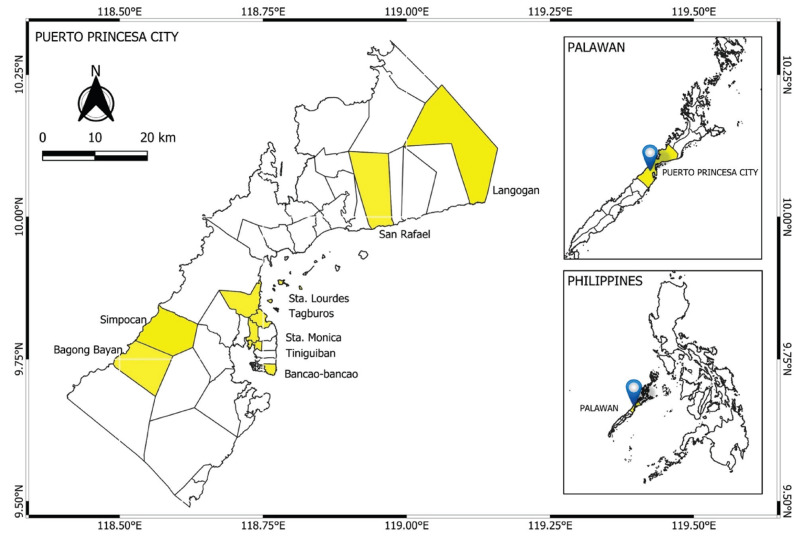
The map of Puerto Princesa City showing the case study sites.

We conducted interviews with 313 adult participants living within 0–200 m from the coastline in both urban (Bancao-bancao, Sta. Monica, Sta. Lourdes, Tagburos, Tiniguiban) and rural villages (Bagong Bayan, Langogan, San Rafael, Simpocan) (Fig. 1). Participants were selected using multi-stage cluster sampling by village, and random sampling of households in the coastal areas. We restricted our participants to those 18 years old and older.

The 0–200 m coastal distance criterion was used to ensure that participants were residents directly exposed to coastal environmental changes and, therefore, more likely to observe and experience climate-related impacts. The sample size of 313 participants provides an approximate ±5–6% margin of error at the 95% confidence level, which is considered adequate for community-based perception studies.

### Survey procedure

The data gathering tool was divided into four sections. The first section collected the socio-demographic profile of the participants, including barangay, sex, age, education, livelihood and monthly income.

The second section assessed participants’ awareness of climate change, perceived threats to family health and safety, and beliefs regarding anthropogenic causes. Responses were measured using a bipolar Likert-type scale, ranging from completely disagree (–2) to completely agree (+2).

The third section focused on participants’ ‘perceived climate change’, specifically SLR, stronger waves, increase in SST and extreme rainfall. For each hazard, respondents compared current conditions with those observed 10 years ago using a five-point bipolar response scale tailored to each indicator. For SLR, responses ranged from more than 1 m seaward from the original shoreline (–2) to more than 1 m inland (+2), with unchanged (original shoreline) coded as 0. For waves, SST and rainfall, response options ranged from –2 (much smaller, much colder, or much less) to +2 (much bigger, much warmer, or much more), with 0 indicating no perceived change.

In this study, ‘perceived climate change’ refers to participants’ observations and experiences of environmental changes, based on their lived experience and local knowledge rather than on direct physical measurements obtained from oceanographic instruments. These perceptions were measured using survey questions that asked participants to compare current conditions with those observed approximately 10 years ago.

The fourth section of the survey assessed respondents’ perceptions of the impacts of climate change on food security. This section included 19 questions (items), each representing aspects of the four pillars of food security: availability (5 items), access (5 items), utilisation (5 items) and stability (4 items). For each item, respondents rated perceived changes in food security conditions compared to 10 years ago. The response scale was a five-point, bipolar Likert-type scale, ranging from −2 (much worse/difficult/decrease) to +2 (much better/improve/increase), with 0 indicating no change. Negative values indicated worsening conditions, positive values indicated improvement, and zero indicated no perceived change. Composite scores for each pillar were then calculated by averaging the responses to the items within that pillar.

The survey questionnaire was developed by a senior faculty member, researchers and students at the College of Fisheries and Natural Sciences of the Western Philippines University in Palawan, Philippines. It was anchored on the Ecosystems-enriched Drivers, Pressures, State, Exposure, Effect, Actions (eDPSEEA) Framework [15] and further informed by the Intergovernmental Panel on Climate Change (IPCC) Special Report on Climate Change and Land [16]. Specifically, the items used to measure the perceived impacts of climate change on food security were adapted from table 5.1 of Chapter 5 of the Special Report on Climate Change and Land Report, which details the interactions between climate drivers and the four pillars of food security – availability, access, utilisation and stability. It was piloted and revised accordingly to ascertain an efficient and effective delivery strategy. It was administered face-to-face in coastal community households utilising the Computer Assisted Personal Interviewing (CAPI) method through the KoBotoolBox Application – a free data gathering toolkit used in remote and challenging locations developed by the Harvard Humanitarian Initiative.

The consent process was conducted at the institutional and household levels. The researchers formally coordinated and solicited the approval of the barangay local government units involved with the study. At the household level, potential participants were informed of the study’s purpose, expected duration and the nature of data that would be collected before getting their permission to join the data collection. They were informed that their participation was voluntary and that there would be no monetary or non-monetary compensation provided. Only the necessary information was gathered, and confidentiality and anonymity were strictly maintained. Before getting their agreement to join, participants were given a chance to ask questions and were informed that they were free to not participate and withdraw their participation during and after the data gathering.

### Data analysis

The data analysis was conducted using Statistical Package for the Social Sciences (SPSS) version 25 for Windows. To provide a complete picture of population characteristics, we used descriptive statistics such as mean, frequency and percentage.

Socio-demographic variables were classified as categorical variables, while climate change variables were classified as continuous variables. Although the climate change variables used Likert items, they were treated as continuous variables, in reference to the five or more categories rule or ‘ordinal approximation of a continuous variable’ [17–20]. Likert-type responses are technically ordinal; however, methodological studies have shown that parametric statistical techniques can be applied when analysing Likert-type data, particularly when the scale contains five or more categories, and the sample size is sufficient [19,21,22]. Parametric statistical tests have been shown to be robust to moderate violations of interval-level assumptions and can produce reliable estimates when applied to Likert responses [19]. In addition, parametric methods may provide greater statistical power and efficiency compared with nonparametric alternatives in many research situations [22].

Regarding the food security pillars, the four composite variables (availability, access, utilisation and stability) were used as outcome variables in the regression analysis. These composite variables were derived from items that outline the complex relationships between food security, food systems and climate change [16]. The items were designed to capture respondents’ subjective perceptions of climate-related changes, consistent with the use of perceptual measures in social research [23]. Before obtaining the summative mean of the Likert items, we ensured internal consistency in each pillar of food security by conducting a reliability test and making sure that the Cronbach alpha value was ≥ 0.70 [24].

The interpretation of the composite Likert mean scores in this study follows a bipolar scale divided into five equal intervals of 0.8 units each. Scores ranging from −2.00 to −1.21 indicate a significant decrease or much worse condition. Scores from −1.20 to −0.41 represent a decrease or worsening of conditions. A score between −0.40 and +0.39 is interpreted as no change. Scores from +0.40 to +1.19 suggest a moderate improvement or better condition, while scores ranging from +1.20 to +2.00 indicate a significant improvement or much better condition. We used multiple linear regression to determine the relationship between socio-demographic variables, climate change impact variables and pillars of food security variables. The same method was used to analyse the relationship between climate change perceptions and the pillars of food security variables.

## Results

### Characteristics of coastal communities in Puerto Princesa City

The participants of the study were 313 participants from coastal communities in Puerto Princesa City (Table 1). The majority of them were males (58%) who were educated at elementary (46%), high school (40%) and college (14%) levels. They were mostly reliant on fisheries and fisheries-related (77%) activities. A majority (54%) of them reside in urban areas, while the rest (46%) live in rural villages. Based on the threshold set by the Philippine Statistics Authority (PSA), 77% of them were poor, while the rest (23%) were not poor.

**Table 1.** The socio-demographic profile of the participants

Variables	Frequency	Percentage
Sex (n = 313)		
Male	181	58
Female	132	42
Age group n = 312)		
1 (19–29)	60	19
2 (30–39)	81	26
3 (40–49)	73	24
4 (50–59)	57	18
5 (60 up)	41	13
Missing data	1	
Education (n = 313)		
Elementary	143	46
High school	126	40
College	44	14
Location classification (n = 313)		
Rural	145	46
Urban	168	54
Livelihood (n = 313)		
Fisheries	241	77
Non-fisheries	72	23
Income level (n = 313)		
Poor	242	77
Not poor	71	23

### Awareness of coastal communities on climate change

**Figure 2:**
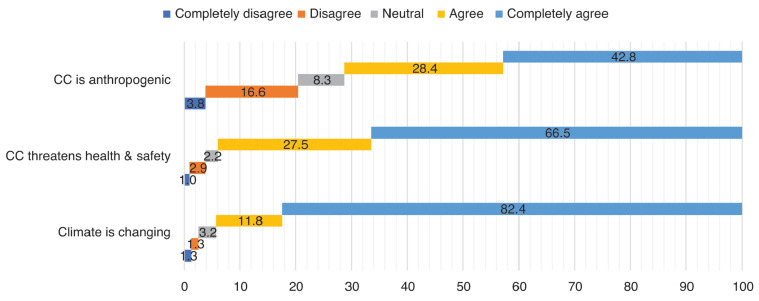
Percentage distribution of perceptions on climate change using a five-point bipolar scale.

The coastal communities in Puerto Princesa City agreed that the climate is really changing (94%) (Fig. 2). Notably, a few were neutral (3%) on the idea and the remaining 3% disagreed. The majority of the participants (94%) believed that the changing climate threatens their personal health and safety, while a few (5%) were either impartial or disagreed. Lastly, many (71%) agreed that climate change was brought about by anthropogenic factors.

### Perceptions of coastal communities on potential impacts of climate change

**Figure 3:**
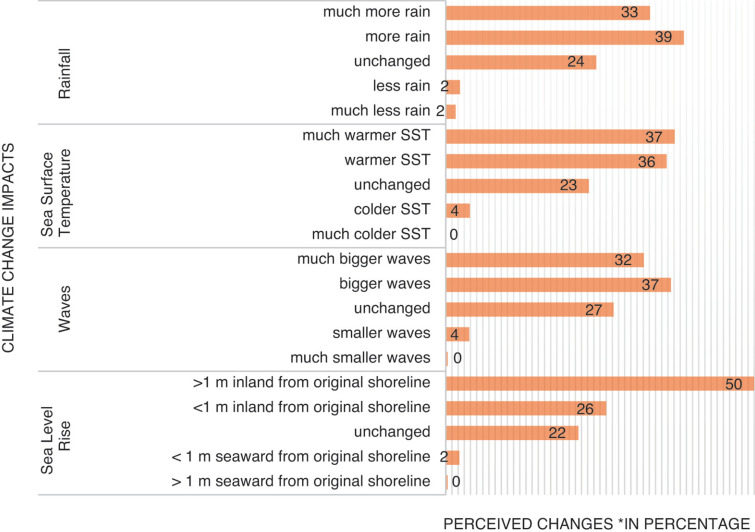
Perceptions of coastal communities on the potential impacts of climate change.

Compared to 10 years ago, coastal communities have observed an inland movement of the shoreline from less to more than 1 m (50% and 26%), likely indicating SLR. These communities also experienced bigger and much bigger (32% and 37%) waves, and warmer and much warmer (36% and 37%) SST. They also apparently have much more (33% and 39%) rainfall compared to observations from 10 years ago (see Fig. 3).

### Perceptions on food security: Puerto Princesa City is less food secure compared to 10 years ago

Coastal communities in Puerto Princesa City have become less food secure compared to 10 years ago (mean = −0.96). They perceived that the available food from the sea had decreased (mean = −1), and that it became difficult to access it (mean = −1.01). Food utilisation practices related to food consumption has been perceived to be worse (mean = −0.97) while the supply itself has become unstable (mean = −0.92) (Table 2).

**Table 2.** Summary of the current perceptions relating to the pillars of food security compared to 10 years ago

Pillars of food security	Mean	Interpretation	Cronbach alpha
Availability			
1. Impact of climate change on overall catch rate	−1.09	Decrease	0.82
2. Impact of climate change on the volume of dominant catch	−1.10	Decrease	
3. Impact of climate change on catch during gleaning and foraging	−0.91	Decrease	
4. Impact of climate change on the fish availability in their community	−0.99	Decrease	
5. Availability of fish/seafoods in their household	−0.92	Decrease	
Score mean	−1.0	Decrease	
Access			
1. Access/capacity to eat a variety of seafoods	−0.98	Difficult	0.75
2. Access to the kinds of seafood you prefer	−0.95	Difficult	
3. Ability (source of income) to purchase seafood/other food	−0.85	Difficult	
4. Effect of food price spikes on their ability to purchase their preferred foods	−1.37	Very difficult	
5. Effect of weather patterns on the timing of fishing activities	−0.97	Difficult	
Score mean	−1.01	Difficult	
Utilisation			
1. Effect of global warming/extreme rainfall on the utilisation of food due to increased risks of vector-borne and infectious diseases	−0.94	Worse	0.78
2. Effect of extreme weather events on coastal pollution	−1.03	Worse	
3. Effect of coastal pollution on nutritional quality and food safety of seafoods	−1.05	Worse	
4. Effect of ocean warming and acidification on fish growth rate and fish quality	−0.98	Worse	
5. Impact of sea surface temperature rise on occurrence of harmful algal blooms, fish kill, coral bleaching and ecosystem degradation	−0.82	Worse	
Score mean	−0.97	Worse	
Stability			
1. Compared to 10 years ago, describe the stability of food supply as impacted by the increased frequency and severity of extreme events?	−0.83	Unstable	0.79
2. Compared to 10 years ago, describe the stability of food supply due to the status of food prices?	−0.96	Unstable	
3. Compared to 10 years ago, describe the stability of food supply due to instability of income?	−0.93	Unstable	
4. Compared to 10 years ago, describe the effect of sea surface temperature to the amount of fish stocks?	−0.94	Unstable	
Score mean	−0.92	Unstable	
Overall mean	−0.96	Less food secure	

### Sex, education, income and location’s contribution to awareness and perception

The multiple linear regression analysis examined how socio-demographic characteristics of coastal community households are associated with their awareness and perceptions of climate change. The analysis considered three outcomes: whether respondents believe the climate is changing, whether climate change threatens health and safety, and whether climate change is anthropogenic. Tests of model assumptions indicated that the requirements of multiple regression were satisfied, with no serious multicollinearity among predictors (variance inflation factor, VIF < 5), approximately normal residuals, and no substantial violations of homoscedasticity.

The regression models explained 2.7% to 12.9% of the variance across the three outcomes (Table 3). The results show that female respondents showed higher agreement that climate change is a threat to health and safety (B = 0.262, *p* < 0.01) than male respondents. Similarly, respondents who are not poor showed higher agreement that climate change is a threat to health and safety (B = 0.212, *p* < 0.05). Education was also associated with perceptions of climate change. Respondents who had reached college level education showed higher agreement that climate change is anthropogenic (B = 1.049, *p* < 0.001). The findings also indicate that respondents residing in urban areas showed lower agreement that climate change is caused by human activities than those living in rural areas (B = −0.459, *p* < 0.001).

**Table 3.** Socio-demographic factors affecting awareness and perception of climate change

Predictors	Climate is changing B (SE)	Climate change threatens health and safety B (SE)	Climate change is anthropogenic B (SE)
Constant	1.706 (0.126)***	1.387 (0.133)***	0.771 (0.207)***
Sex			
(Ref = Male)			
Female	−0.019 (0.088)	0.262 (0.093)**	0.062 (0.145)
Education			
(Ref = Elementary)			
High School	−0.055 (0.090)	0.015 (0.095)	0.223 (0.147)
College	0.065 (0.139)	0.202 (0.148)	1.049 (0.229)***
Livelihood			
(Ref = Fisheries)			
Non-fisheries)	0.087 (0.110)	−0.084 (0.116)	−0.100 (0.180)
Age group			
(Ref = 18–29)			
30–39	0.073 (0.121)	0.097 (0.128)	0.173 (0.198)
40–49	0.046 (0.123)	0.065 (0.131)	0.325 (0.203)
50–59	−0.190 (0.136)	−0.127 (0.145)	0.020 (0.224)
60 up	0.081 (0.147)	0.135 (0.156)	0.017 (0.241)
Income			
(Ref = Poor)			
Not poor	−0.031 (0.097)	0.212 (0.103)*	0.064 (0.160)
Location			
(Ref = Rural)			
Urban	0.044 (0.081)	−0.076 (0.086)	−0.459 (0.134)***
Model fit statistics			
R^2^	0.027	0.070	0.129
Significance	0.587	0.014	0.000

*p < 0.05; **p < 0.01; ***p < 0.001.

### Sex, age, income and location’s contribution to perceptions on food security

The regression analysis also highlighted several important findings on how the socio-economic profile may influence the perceived status of food security of coastal communities when compared to 10 years ago (Table 4). The models explained 14.1% to 17.4% of the variance in food availability, access and utilisation, while the model for food stability explained a smaller proportion of variance (R^2^ = 0.049).

**Table 4.** Socio-demographic factors affecting the perception of food security

Predictors	Availability B (SE)	Access B (SE)	Utilisation B (SE)	Stability B (SE)
Constant	−1.18 (0.11)***	−1.15 (0.09)***	−0.93 (0.09)***	−1.0 (0.10)***
Sex				
(Ref = Male)				
Female	−0.007 (0.076)	0.009 (0.061)	0.122 (0.059)**	0.032 (0.071)
Education				
(Ref = Elementary)				
High School	−0.004 (0.077)	0.072 (0.062)	0.058 (0.059)	0.103 (0.072)
College	−1.47 (0.120)	0.035 (0.062)	−0.037 (0.094)	0.116 (0.112)
Livelihood				
(Ref = Fisheries)				
Non-fisheries	0.109 (0.094)	0.074 (0.076)	0.037 (0.074)	0.064 (0.088)
Age group				
(Ref = 18–29)				
30–39	0.034 (0.104)	−0.023 (0.083)	−0.003 (0.081)	−0.077 (0.097)
40–49	−0.066 (0.106)	−1.35 (0.085)	−0.014 (0.083)	0.016 (0.099)
50–59	−0.221 (0.117)	−0.083 (−0.062)	0.177 (0.092)*	−0.003 (0.109)
60 up	−0.150 (0.126)	−1.37 (0.101)	0.103 (0.099)	−0.055 (0.117)
Income				
(Ref = Poor)				
Not poor	−0.178 (0.084)*	−1.77 (0.067)**	−0.301 (0.066)***	−0.201 (0.078)
Location				
(Ref = Rural)				
Urban	0.519 (0.070)***	0.357 (0.056)***	−0.156 (0.055)**	0.120 (0.065)*
Model fit statistics				
R^2^	0.174	0.145	0.141	0.049
Significance	0.000	0.000	0.000	0.117

*p < 0.05; **p < 0.01; ***p < 0.001.

The results indicate that respondents who are not poor perceived that food availability has decreased (B = −0.178, *p* < 0.05), access to food has become more difficult (B = −0.177, *p* < 0.01), and food utilisation has worsened (B = −0.301, *p* < 0.001) compared with conditions 10years ago. Another notable finding is that female respondents (B = 0.122, *p* < 0.01) and those aged 50–59 years (B = 0.177, *p* < 0.05) perceived there are improvements in food utilisation. Location also played an important role in shaping perceptions of food security. Respondents residing in urban coastal communities reported improvements in food availability (B = 0.519, *p* < 0.001), access (B = 0.357, *p* < 0.001) and stability (B = 0.120, *p* < 0.05) compared with those living in rural coastal areas. However, despite these perceived improvements, urban respondents also reported worsening conditions in food utilisation (B = −0.156, *p* < 0.01) compared with rural areas.

### Climate change may affect food security

Finally, the analysis examined how perceived climate change hazards are associated with the four pillars of food security: availability, access, utilisation and stability. The results (Table 5) show that perceived SLR is negatively associated with food availability (B = −0.097, *p* < 0.05), food access (B = −0.107, *p* < 0.05), food utilisation (B = −0.081, *p* < 0.05) and food stability (B = −0.113, *p* < 0.01). Perceived increase in SST is significantly linked with poorer food utilisation (B = −0.115, *p* < 0.01) and reduced food stability (B = −0.064, *p* < 0.05). The models explain 2.8% to 7.2% of the variance across the four food security pillars.

**Table 5.** Perceived impacts of climate change on food security

Predictors	Availability	Access	Utilisation	Stability
Constant	−0.938 (0.066)***	−0.921 (0.061)***	−0.777 (0.058)***	−0.718 (0.007)***
Sea level rise	−0.097 (0.040)*	−0.107 (0.037)*	−0.081 (0.035)*	−0.113 (0.040)***
Waves	−0.002 (0.041)	0.009 (0.036)	0.001 (0.036)	−0.008 (0.041)
Sea surface temperature	−0.011 (0.038)	−0.021 (0.036)	−0.115 (0.034)***	−0.064 (0.039)
Rainfall	0.047 (0.037)	0.049 (0.034)	0.035 (0.032)	0.011 (0.037)
Model fit statistics				
R^2^	0.028	0.038	0.072	0.051
Significance	0.066	0.018	0.000	0.003

*p < 0.05; **p < 0.01; ***p < 0.001.

## Discussion

This paper seeks to understand the characteristics of the coastal communities in a city within an LMIC country and further look at their awareness and perception of climate change, food security, and in return look into how the subtexts of these phenomena along with their exposure to the changing situation may potentially be linked to their respective perception and awareness. Perception and awareness are two of the essential components to possibly identify adaptive capacity initiative. Collective local and traditional knowledge of coastal communities gained through years of exposure and experiences is critical information that will help shape a sound and bespoke climate change mitigation and adaptation policy and initiatives [25].

### Characteristics of coastal communities in Puerto Princesa City

To better understand the attributes of coastal communities of Puerto Princesa City, this study looked at several factors, including the respondents’ sex, age group, education, location, livelihood and income level.

Some of the notable findings described in this study highlight that (1) most of the respondents were not able to pursue a college degree, (2) the location of their household within the city’s jurisdiction showed a marginal difference between urban and rural settings, (3) their livelihood is mostly fisheries-related and (4) most of them are categorised as ‘poor’. Poor households in coastal communities are vulnerable to the impacts of climate change [26]. Compounded with exposure to the changing climate, these coastal households are at an even higher vulnerability index [27]. Poverty and vulnerability are interwoven in such a way that a gap in development can extremely aggravate the impacts of climate change and, in return, continuously impair the adaptive capacity of mostly vulnerable regions like the Philippines [16].

### High awareness of the occurrence and impact of climate change

Climate change is an urgent global issue with far-reaching implications, and coastal communities are often at the forefront of its impacts [28,29]. The result of this study highlights a significantly high consensus among participants that human-induced climate change is perceived in coastal communities in Puerto Princesa City, and that residents recognise its potential impact on their health and safety. Coastal residents are often the first to observe changes in their environment. This level of awareness is higher compared to other coastal communities in the Philippines, such as Cotabato City (23%) [30], other areas in the MIMAROPA Region (65.4%) and the national average (40.1%) [31]. This could be linked to several factors, which include observation of noticeable shifts in the local environment and temperature [31], and exposure to recent memorable climate events [32]. In Southeast Asia, climatic events and hazards in the past 5 years, characterised by cyclones, SLR, rainfall and flooding, among others [33], make coastal communities more vulnerable compared to other settings. Often engaged in fishing, these communities are also the ones who are potentially at risk for health issues, psychological stress and reduced productivity linked to a physically demanding occupation [34] in a warmer work environment [35].

### The shore is getting closer inland

One of the notable observations by the coastal communities is the movement of the shoreline inland, a manifestation linked to SLR [36] and a potential predictor of climate change [37]. This collective perception in Puerto Princesa City could also be associated to the reported rise of the mean sea level in the Philippines ranging from 5.7 to 7 mm/year, particularly in Puerto Princesa City, reportedly at 6.19 mm/year [38] – both more or less double the world’s average reported by NASA at 3.4 mm/year. It is also important to note that the sea level is rising faster in the west compared to the east of the Pacific [39] posing even more risks to the developing countries in the Western Pacific region, like the Philippines.

This study showed that observation of the movement of the shoreline inland and the perceived increase in SST are linked to food insecurity in the coastal communities in this setting. Many scenarios may contribute to this occurrence. This may be attributed to the changes in the location of fishing grounds, fish migration and increased fishing days, potentially compromising fish quality and quantity, while increasing the fishing input cost [32], which could further increase the probability of food insecurity of fishing and fish-dependent coastal communities.

### Coastal communities in the Western Pacific are becoming less food secure

Coastal communities in Puerto Princesa City have observed decreased seafood availability. This could be explained by the study of Palla et al. [34], who reported on the low well-being of the fish in Palawan beginning in 2006, that was still steadily declining along with the same trend in wild fishing in the Philippines and the world [35]. This could be an added explanation for the much more difficult access to seafood and other food experienced by these coastal communities. This trend may continue to occur in the central west part of the Pacific and could result in even greater reductions in fish catch and income among fishers [40].

This decreasing seafood availability may put coastal communities at risk. First, this could potentially negatively impact the household income of the fishers [41,42]. Livelihood and income are predictors of food security [43]. Decreased income is associated with food insecurity in coastal fishing communities in Africa [44], a situation not far from that of the coastal communities in Puerto Princesa City and in the Western Pacific where coastal fishers were observed to sell fresh catch to buy other food items [45], including ultra-processed food.

Second, this could plausibly reduce the protein source of the coastal communities. Through the years, individual Filipinos’ average seafood consumption estimates have declined from 36 kg/year in 1993 to 14.34 kg in the 2018–19 as shown in the Food and Nutrition Research Institute Expanded National Nutrition Survey [46]. Now, a typical Filipino household whose income comes from fishing consumes 0.48 kg of fish per day. This finding is particularly lower in poor households, as increasing wealth is associated with increasing access to fish [45,47] and vice versa. Aside from eating more fish, households that have more wealth tend to eat less processed food, such as tinned and salted fish. Despite the assumption that fish is available for those who produce, those who do are among the most seriously malnourished [48].

Most of the study participants from the coast of Puerto Princesa City are poor. Poor households are dependent on fish and shellfish for their protein and micronutrient needs [47,48], a crucial building block for children aged 1–5 years old and those in energy-demanding occupations in coastal communities such as fishing and gleaning. Poor households also tend to consume low-value fish as a cheap source of animal protein [47]. If poor households have reduced access to food compared to 10 years ago as shown by the results, there will be a more serious impact on both their nutrition and economic status [48].

Price is a significant predictor of access to food [49], and to combat food insecurity, food should not only be available, but it should also be accessible [49]. These phenomena not only impact the economic and physical health and well-being of the coastal communities themselves but could also negatively impact the mental health of small-scale fishers, as shown by the study conducted in Ghana among small-scale fishers [50]. Improving household assets and knowledge through bridging both economic and educational gaps, providing other fisheries options and stabilising the market that is at par with the purchasing power of the coastal households is critical to enabling food-secure communities [45,47].

### Food has also become less available, less accessible and less safe for the non-poor

Many studies in India [51], Kenya [52] and the United States [53] highlight that increased income is associated with food security in its general sense. However, the result of this study provides us with a new lens that highlights the probability that non-poor households in LMICs also experience less food availability, accessibility and safety. There are rare cases where high-income households also report food insecurity but, in this setting, this scenario can be associated with the high inflation rate in the Philippines during the period of the study, which peaked at 8.1% at the end of 2022, the highest since November 2008 [54]. The Philippine Senate particularly reported that the price of fish and other seafood rose by 6.7% during the same period [55].

Inflation and the price of goods can be associated with increased food insecurity not only in LMICs but other high-income countries [56,57]. And this, in many areas of the world, including LMICS, is likely driven by the impacts of the changing climate [58,59] and other compounding factors.

### Urban coastal communities have better food availability, access and stability

Geography includes another layer of elements in food security, which the findings of this study highlight. Studies suggest that there is a need to look deeply into the relationship between urban and rural communities in terms of food production and consumption [60]. In this area of the Western Pacific, our study highlights that urban communities tend to have an increasing level of food availability, access and stability compared to their rural counterparts. In this setting, most of the rural fishers, due to fear of spoilage as a result of not having access to electricity and distribution capacity, tend to sell their catch to local buyers and middlemen. These middlemen and local buyers sell the catch in the urban centres and cities. Compared to rural areas, there are also more vendors, sellers, markets, stalls and supermarkets in urban centres and cities [56,57]. This supports the studies in nearby settings which suggest that production does not correlate to consumption [61].

In a study in Indonesia [62], the rural communities also link their difficulty in being food secure to their dependence on the natural ecosystems. Coastal communities in this part of the Western Pacific, as the result suggests, are heavily dependent on fishing. This, therefore, makes them more susceptible to food insecurity brought about by fluctuating climate, and crop failure or less catch. This study further points out that well-maintained nature and local knowledge influence food security.

## Conclusions

The coastal communities have a high level of climate change awareness, and they believe that this threatens their health and safety. Coastal communities observed that the original shoreline is moving closer inland, waves are bigger, SST is warmer and the amount of rainfall increased. There is a decrease in the volume of fish caught, catch rate in wild fishing, catch rate in gleaning and availability of food in fish supply to both the community population and households. Food stability is affected by the perceived severity of extreme events, spikes in food prices, income from fishing activities and fish stocks. Access to a variety of food and preferred seafood has become difficult. The ability to purchase, the spike in food prices and the changing weather have made it difficult to access food. ‘Not poor’ communities have significantly lower levels of food availability, access and utilisation. Urban coastal communities have significantly higher food availability, access and stability. SLR is a significant predictor of lower/decreased food access, utilisation and stability. An increase in SST is a significant predictor of the lower/decreased utilisation of resources towards food security.

### Policy implications of the study

This study calls for stronger policies distribution of government services and climate change-related intervention with a clear lens of equity, particularly in geographically isolated and vulnerable regions, with strengthened coordination between the national and local governments. This also suggests a reframed climate change mitigation strategy with food security explicitly as one of its pillars, not only creating systems for sustained production and diverse sources but also for equitable food distribution and access among the population. This also strongly appeals for structural and systemic support for nature-dependent coastal communities whose food, nutrition and livelihood are intrinsically linked to the health of the ocean and the impacts of climate change.

Future research on climate change should focus on how to capitalise on the coastal community’s awareness and increase their inclusion in the development and implementation of climate change adaptation initiatives. Government units should further utilise local ecological knowledge and perception, creating bespoke climate change adaptation and food security initiatives.

### Limitations of the study

While this study endeavours to contribute valuable insights into the impact of climate change on the food security of coastal communities in Puerto Princesa City, it is essential to acknowledge certain limitations that may impact the robustness and generalisability of the findings. This includes response bias and guiding effect where most of the respondents are elementary school graduates and may not fully understand some technical words. Additionally, most of them are not well-read and do not know about scientific events. This resulted in the survey questionnaire administrator sometimes guiding the participant to understand the context of the question, which can introduce response bias and affect the authenticity of the collected data. Obtaining unbiased and authentic responses is important, but maintaining complete neutrality during data collection is challenging and can have implications for the validity of the results.

## Data Availability

The datasets generated during and/or analysed during the current study are available from the corresponding author on reasonable request.

## References

[1] Stern DI, Kaufmann RK (2014). Anthropogenic and natural causes of climate change. Clim Change [online].

[2] Tol RSJ (2018). The economic impacts of climate change. Rev Environ Econ Policy [online].

[3] Paun A, Acton L, Chan WS (2018). Fragile Planet: Scoring climate risks around the world.

[4] World Bank Group (2022). Country climate and development report: Philippines. [online].

[5] Wheeler T, von Braun J (2013). Climate change impacts on global food security. Science [online].

[6] Concern Worldwide, Welthunherhilfe (2024). Global Hunger Index 2024: Philippines.

[7] IBON Foundation (2024). IBON [Non Government organization]. PH food insecurity worsening to among highest in region underscores multiple gov’t failures – IBON. [online].

[8] Integrated Food Security Phase Classification (2015). Integrated Food Security Phase Classification: evidence and standards for better food security. [online].

[9] Javier C, Ducay AJD, Duante CA, Desnacido JP, Misagal MaEB, Vargas MB (2024). Diet adequacy and food cost of food-secure and insecure households: evidence from the 2018 ENNS. Philipp J Sci [online].

[10] Philippine Statistics Authority (2023). 2023 [N03] Official poverty statistics of the Philippines among the basic sectors in the Philippines. [online].

[11] Capili EB, Ibay ACS, Villarin JRT (2005). Proceedings of OCEANS 2005 MTS/IEEE. [online].

[12] Milfont TL, Evans L, Sibley CG, Ries J, Cunningham A (2014). Proximity to coast is linked to climate change belief. PLoS One [online].

[13] City Government of Puerto Princesa (2020). City of Puerto Princesa Comprehensive Development Plan 2020–2025. [online].

[14] Regoniel P, Macasaet MT, Mendoza N (2017). Economic analysis of climate change adaptation options in Honda Bay, Puerto Princesa, Philippines. [online].

[15] Reis S, Morris G, Fleming LE, Beck S, Taylor T, White M (2015). Integrating health and environmental impact analysis. Public Health [online].

[16] Intergovernmental Panel on Climate Change (IPCC) (2023). Climate change 2022 – impacts, adaptation and vulnerability: Working Group II Contribution to the Sixth Assessment Report of the Intergovernmental Panel on Climate Change. [online].

[17] Johnson DR, Creech JC (1983). Ordinal measures in multiple indicator models: a simulation study of categorization error. Am Sociol Rev [online].

[18] Norman G (2010). Likert scales, levels of measurement and the “laws” of statistics. Adv Health Sci Educ Theory Pract [online].

[19] Sullivan G, Artino A (2013). Analyzing and interpreting data from Likert-type scales. J Grad Med Educ [online].

[20] Zumbo BD, Zimmerman DW (1993). Is the selection of statistical methods governed by level of measurement?. Can Psychol [online].

[21] Huh I, Gim J (2025). Exploration of Likert scale in terms of continuous variable with parametric statistical methods. BMC Med Res Methodol [online].

[22] DeWees TA, Mazza GL, Golafshar MA, Dueck AC (2020). Investigation into the effects of using normal distribution theory methodology for Likert scale patient-reported outcome data from varying underlying distributions including floor/ceiling effects. Value Health [online].

[23] Collins KMT, Onwuegbuzie AJ, Jiao QG (2007). A mixed methods investigation of mixed methods sampling designs in social and health science research. J Mix Methods Res [online].

[24] Sharma B (2016). A focus on reliability in developmental research through Cronbach’s Alpha among medical, dental and paramedical professionals. Asian Pac J Health Sci [online].

[25] Dolan AH, Walker IJ (2004). Understanding vulnerability of coastal communities to climate change related risks. J Coast Res [online].

[26] Asuero MP, Nelson GLM, Espaldon MVO, Acosta-Michlick LA, Macandog DM, Lalican NM (2012). Social characteristics and vulnerabilities of disaster-prone communities in Infanta, Quezon, Philippines. J Environ Sci Manag.

[27] Ehsan S, Ara Begum R, Abdul Maulud KN (2022). Household external vulnerability due to climate change in Selangor coast of Malaysia. Clim Risk Manag [online].

[28] Nicholls RJ, Cazenave A (2010). Sea-level rise and its impact on coastal zones. Science [online].

[29] Barnard PL, Short AD, Harley MD, Splinter KD, Vitousek S, Turner IL (2015). Coastal vulnerability across the Pacific dominated by El Niño/Southern oscillation. Nat Geosci [online].

[30] Husain JM (2022). Perceived impacts of climate change, vulnerability, and fisherfolks’ choices of adaptation practices in Liguasan Marshlands. PhD thesis.

[31] Bollettino V, Alcayna-Stevens T, Sharma M, Dy P, Pham P, Vinck P (2020). Public perception of climate change and disaster preparedness: evidence from the Philippines. Clim Risk Manag [online].

[32] Damasio L, Peninno MG, Lopes P (2020). Small changes, big impacts: geographic expansion in small-scale fisheries. Fish Res [online].

[33] Noor NM, Abdul Maulud KN (2022). Coastal vulnerability: a brief review on integrated assessment in Southeast Asia. J Mar Sci Eng [online].

[34] Palla HP, Pagliawan HB, Rodriguez EF, Montaño BS, Cacho G, Gonzales BJ (2018). Length-weight relationship of marine fishes from Palawan, Philippines. Palawan Sci [online].

[35] Pauly D, Zeller D (2017). Comments on *FAOs State of World Fisheries and Aquaculture* (SOFIA 2016). Mar Policy [online].

[36] Cazenave A, Cozannet GL (2014). Sea level rise and its coastal impacts. Earths Future [online].

[37] Elneel L, Zitouni MS, Mukhtar H, Galli P, Al-Ahmad H (2024). Exploring key aspects of sea level rise and their implications: an overview. Water [online].

[38] Oscar M (2018). Lopez Center. Philippine climate change assessment report. Sci Clim Resilient Communities [online].

[39] Peyser CE, Yin J, Landerer FW, Cole JE (2016). Pacific sea level rise patterns and global surface temperature variability. Geophys Res Lett [online].

[40] Biswas BK, Svirezhev YuM, Bala BK, Wahab MA (2009). Climate change impacts on fish catch in the world fishing grounds. Clim Change [online].

[41] Anticamara JA, Go KTB (2016). Spatio-temporal declines in Philippine fisheries and its implications to coastal municipal fishers’ catch and income. Front Mar Sci [online].

[42] Danquah JA, Roberts CO, Appiah M (2021). Effects of decline in fish landings on the livelihoods of coastal communities in Central Region of Ghana. Coast Manag [online].

[43] Weldemariam LF, Sakdapolrak P, Ayanlade A (2023). Household food-security strategies and migration in Tigray, Northern Ethiopia. Sci Afr [online].

[44] Muringai RT, Mafongoya PL, Lottering R (2021). Climate change and variability impacts on Sub-Saharan African fisheries: a review. Rev Fish Sci Aquac [online].

[45] Cruz-Trinidad A, Aliño PM, Geronimo RC, Cabral RB (2014). Linking food security with coral reefs and fisheries in the coral triangle. Coast Manag [online].

[46] Lowe J, Souther D, Muallil R, Angeles-Adgeppa I, Goyena EA, Javier CA (2022). The state of fish in nutrition systems in the Philippines. [online].

[47] Mohan Dey M, Rab MA, Paraguas FJ, Piumsombun S, Bhatta R, Ferdous Alam M (2005). Fish consumption and food security: a disaggregated analysis by types of fish and classes of consumers in selected Asian countries. Aquac Econ Manag [online].

[48] Kent G (1997). Fisheries, food security, and the poor. Food Policy [online].

[49] Beveridge MCM, Thilsted SH, Phillips MJ, Metian M, Troell M, Hall SJ (2013). Meeting the food and nutrition needs of the poor: the role of fish and the opportunities and challenges emerging from the rise of aquaculture. J Fish Biol [online].

[50] Freduah G, Fidelman P, Smith TF (2017). The impacts of environmental and socio-economic stressors on small scale fisheries and livelihoods of fishers in Ghana. Appl Geogr [online].

[51] Maitra C, Rao DSP (2015). Poverty–food security nexus: evidence from a survey of urban slum dwellers in Kolkata. World Dev [online].

[52] Olielo T (2013). Food security problems in various income groups of Kenya. Afr J Food Agric Nutr Dev [online].

[53] Walker RJ, Garacci E, Dawson AZ, Williams JS, Ozieh M, Egede LE (2021). Trends in food insecurity in the United States from 2011–2017: disparities by age, sex, race/ethnicity, and income. Popul Health Manag [online].

[54] Senate Economic Planning Office (2023). Inflation in 2022: At A Glance [Senate Report].

[55] Alcantara LB, Creencia LA, Madarcos JRV, Madarcos KG, Jontila JBS, Culhane F (2023). Climate change awareness and risk perceptions in the coastal marine ecosystem of Palawan, Philippines. UCL Open Environ [online].

[56] Coleman-Jensen A, Gregory CA (2014). Prevalence of U.S. food insecurity is related to changes in unemployment, inflation, and the price of food.

[57] Jacobs P Household food insecurity, rapid food price inflation and the economic downturn in South Africa. Agenda Empower Women Gend Equity.

[58] Morris PM, Neuhauser L, Campbell C (1992). Food security in rural America: a study of the availability and costs of food. J Nutr Educ [online].

[59] Sibanda L, Blottnitz HV (2018). Urban food systems governance and poverty in African Cities. [online].

[60] Gebre T, Gebremedhin B (2019). The mutual benefits of promoting rural–urban interdependence through linked ecosystem services. Glob Ecol Conserv [online].

[61] Brun V, Celis AIJ, Dubrana C, Saludsod MaM, Creencia LA, Gajardo LJA (2025). From ocean to markets: fish exports threaten nutrition security in coastal communities. Commun Earth Environ [online].

[62] Yusriadi Y, Cahaya A (2022). Food security systems in rural communities: a qualitative study. Front Sustain Food Syst [online].

